# Case Report: Steroid-Responsive Takotsubo Cardiomyopathy Associated With Cytokine Storm and Obstructive Shock

**DOI:** 10.3389/fcvm.2022.931070

**Published:** 2022-07-11

**Authors:** Brent Gudenkauf, Michael R. Goetsch, Rachit M. Vakil, Oscar Cingolani, Luigi Adamo

**Affiliations:** ^1^Department of Medicine, Johns Hopkins University School of Medicine, Baltimore, MD, United States; ^2^Division of Cardiology, Department of Medicine, Johns Hopkins University School of Medicine, Baltimore, MD, United States

**Keywords:** stress cardiomyopathy, Takotsubo stress cardiomyopathy, heart failure, cytokine storm, obstructive shock

## Abstract

A growing body of evidence suggests that inflammation may play a key role in the development of Takotsubo stress cardiomyopathy. Here, we report the case of a 63-year-old woman who presented with chest pain and was diagnosed with this cardiomyopathy. After an initial improvement, the patient experienced a systemic inflammatory response of unclear origin and deteriorated rapidly into obstructive shock. Her presentation was considered consistent with cytokine storm. She was, therefore, treated with steroids with rapid improvement in her clinical picture. She relapsed after the taper. Endomyocardial biopsy soon after initiation of pulse dose steroids showed macrophage and lymphocytic infiltration. This case highlights the potential intimate connection between systemic inflammatory response and Takotsubo stress cardiomyopathy and contributes to the evolving understanding of inflammation in the pathogenesis of this disease.

## Introduction

Takotsubo stress cardiomyopathy is classically a transient, acute form of heart failure characterized by apical hypokinesis and hyperdynamic basal segments in the absence of coronary artery disease (1). It frequently occurs after an identifiable stressor, and it has been postulated that the pathogenic mechanism centers on catecholamine surge and resultant myocardial toxicity/dysfunction (2). However, one of the histologic hallmarks of stress cardiomyopathy is macrophage and lymphocytic infiltration of the myocardium (2, 3), and there are emerging data from both human studies and animal models indicating that inflammation plays a key role in the development of this syndrome (2–9). Recently, analysis of cardiotoxicity triggered by the rapid release of cytokines that occurs with cytokine release syndrome after CAR-T cell therapy has provided robust evidence that rapid elevation in the serum levels of inflammatory cytokines is associated with acute left ventricular dysfunction with Takotsubo features (10–12). The presence of a systemic inflammatory response at the time of diagnosis of Takotsubo stress cardiomyopathy has also been shown to be a negative prognostic factor. However, the role that systemic inflammation plays in the evolution of Takotsubo and the potential role of anti-inflammatory therapies in Takotsubo remain unknown (13). Here, we report a case of Takotsubo stress cardiomyopathy that developed a systemic inflammatory response, cytokine storm, and hemodynamic collapse after initial presentation, which responded swiftly to pulse dose steroids. This case is unique as it provides insight into the role that systemic inflammation might play in the pathogenesis of this cardiomyopathy, and it highlights the potential role of anti-inflammatory drugs in the treatment of life-threatening hemodynamic compromise secondary to Takotsubo.

## Case Description

A 63-year-old woman presented to the emergency department with acute chest pain. The patient had well-controlled type 2 diabetes mellitus, hyperlipidemia, chronic migraines, anxiety, depression, gastroesophageal reflux disease, splenic and renal artery aneurysms, a right ovarian cyst, and ankylosing spondylitis (and she was on chronic immunosuppression for this). She was status post-cholecystectomy, bilateral saline breast implants, chin reduction, upper eye and facelift, and abdominoplasty.

She was in her usual state of health until that day when she developed aching deep chest pain, dyspnea, and migraine while at a grandchild's baseball game. She denied any strong emotions provoked by the baseball game itself and had no other current emotional stressors in her life. On arrival, she was tachycardic, with a regular rate at 148 beats/min, with a respiratory rate in the 20 s; blood pressure, 98/68 mmHg; and oxygen saturation, 98% breathing ambient air. Her jugular vein was distended, but examination of the cardiovascular, pulmonary, and other systems was otherwise normal.

Cross-sectional imaging excluded pulmonary embolism and aortic dissection. An electrocardiogram showed sinus tachycardia and ST-elevations in inferior leads. Troponin I was 14.1 ng/ml (reference range, RR, <0.4 ng/ml). The differential diagnosis of her presentation included takotsubo cardiomyopathy and acute coronary syndrome. She was, therefore, urgently transferred to the cardiac catheterization laboratory. A coronary angiogram showed no coronary disease. The left ventriculogram showed reduced ejection fraction (LVEF) to 20% and severe hypokinesis of the mid- and distal anterior, inferior, and apical walls with sparing of the base. Transthoracic echocardiogram showed normal left ventricular size; ejection fraction, 30%; and akinetic apical lateral/anterior/inferior and mid anterior septal/lateral wall segments with an aneurysmal appearance of the left ventricular apex. She was admitted to the cardiac care unit with a diagnosis of Takotsubo stress cardiomyopathy.

On admission, her white blood cell count (WBC) was 16,960/cubic mm, the erythrocyte sedimentation rate (ESR) was 8 mm/h (RR, 4–30 mm/h), and C-reactive protein (CRP) was 10.3 mg/dL (RR <0.5 mg/dL). The patient developed a fever on Hospital Day 1, which remained despite broad-spectrum antibiotics. Infectious workup was negative (Table 1). She initially improved, but, on Hospital Day 5, she developed hypotension and recurrence of lactic acidosis (Table 2). Of note, the patient was not taking metformin prior to admission or while hospitalized. There was no significant left ventricular outflow tract (LVOT) gradient upon admission, but the patient developed a gradient of 61 mmHg at this time. A pulmonary artery catheter was placed and showed a mean right atrial pressure of 14 mmHg, mean pulmonary arterial pressure of 21 mmHg, mean pulmonary capillary wedge pressure of 18 mmHg, mixed venous oxygen saturation of 47%, cardiac output (CO) of 4.32 L/min, cardiac index (CI) of 2.5 L/min/m^2^, pulmonary vascular resistance of 0.69 Woods units, and indexed systemic vascular resistance of 1,939 dynes-s/cm^5^/m^2^. She was started on phenylephrine to maintain cardiac output in the context of LVOT obstruction.

**Table 1:** Laboratory investigations.

**Laboratory Study**	**Result**	**Reference range**
Cardiac troponin	**14.1 ng/mL on admission, to 0.07 ng/mL**	<0.04 ng/mL
Pro-B-type natriuretic peptide	**3,327 pg/mL**	<125 pg/mL
White blood cell count	**16,960 cells/mL**	4,500–11,000 cells/mL
Hemoglobin	14.5 g/dL	12.0–15.0 g/dL
Creatinine	0.6 mg/dL	0.5–1.2 mg/dL
Antinuclear antibody	Negative	Negative
C3 complement	88.9 mg/dL	81–157 mg/dL
C4 complement	20.05 mg/dL	13–39 mg/dL
Interleukin 6	**94.8 downtrending to 5.1 pg/mL**	<10 pg/mL
Interleukin 2 receptor	**2,344 pg/mL**	532–1,891 pg/mL
Serum IgA	181 mg/dL	61–348 mg/dL
Serum IgM	73 mg/dL	35–242 mg/dL
Serum IgE	110 kU/L	<114 kU/L
Serum IgG	557 mg/dL	610–1,616 mg/dL
Serum IgG_1_	304 mg/dL	382–929 mg/dL
Serum IgG_2_	129 mg/dL	242–700 mg/dL
Serum IgG_3_	38.3 mg/dL	21.8–176.1 mg/dL
Serum IgG_4_	2.3 mg/dL	3.9–86.4 mg/dL
Erythrocyte sedimentation rate	**57 downtrending to 30 mm/hr**	4–30 mm/hr
C-reactive protein	**10.3 mg/dL**	<0.5 mg/dL
Bacterial blood cultures	Negative	Negative
Fungal blood cultures	Negative	Negative
Urine cultures	Negative	Negative

*Abnormal values are in bold*.

**Table 2:** Case timeline.

**Time**	**Events**
Day 1	Patient presented with chest pain. Electrocardiogram showed ST-segment elevation in the inferior leads.
Day 2	Coronary angiogram showed no obstructive coronary disease and left ventriculogram showed apical ballooning. Transthoracic echocardiogram showed EF 20%. The patient was admitted to the CCU. Due to newly developed fever and hypotension, infectious workup commenced, and antibiotics were started for presumed mixed shock.
Day 3	Lactic acidosis resolved. The pulmonary artery catheter was removed.
Day 5	Transthoracic echocardiogram showed new left ventricular outflow tract gradient of 30 mmHg. Hypotension developed, with recurrence of lactic acidosis.
Day 6	Transthoracic echocardiogram showed increased left ventricular outflow tract gradient to 61 mmHg.
Day 7	Right heart catheterization showed mildly elevated filling pressures and cardiac index of 2.5 L/min/m^2^. Phenylephrine was started.
Day 7	Due to ongoing fevers and elevated inflammatory markers, methylprednisolone was initiated.
Day 8	Lactic acidosis resolved.
Day 9	Transthoracic echocardiogram showed resolution of left ventricular outflow tract gradient and EF 35%. Phenylephrine was weaned off.
Day 11	Endomyocardial biopsy was performed, compatible with Takotsubo cardiomyopathy. Right heart catheterization showed mildly elevated filling pressures and cardiac output of 5.5 L/min. Steroid taper commenced. Antibiotics were discontinued.
Day 12	Cardiac magnetic resonance imaging showed distal mid-cavity to apical hypokinesis with a hyperdynamic base, without edema or late gadolinium enhancement.
Day 15	Transthoracic echocardiogram showed EF 55–60%. The patient was discharged from the hospital on metoprolol and prednisone.
Day 28	The patient took her final dose of prednisone.
Day 29	The patient presented again with sharp, positional chest pain. She was admitted for pericarditis.
Day 31	Transthoracic echocardiogram showed recurrence of apical and apical-lateral segment hypokinesis, with EF 65%. Aspirin and colchicine were started.
Day 35	Pain remitted and the patient was discharged from the hospital.
4 months after initial admission	The patient was seen in the clinic and doing well, without further chest pain, or symptoms/signs of heart failure. NSAID was stopped and colchicine was continued.

On the day of her decompensation, her ESR was 57 mm/h and CRP was 17.2 mg/dL. Ferritin was 1,691 ng/ml, serum interleukin-6 (IL-6) was 94.8 pg/ml (RR < 10 pg/ml), and soluble interleukin-2 (IL-2) receptor was 2,344 pg/ml (RR, 532–1,891 pg/ml).

## Management

**Figure 1:**
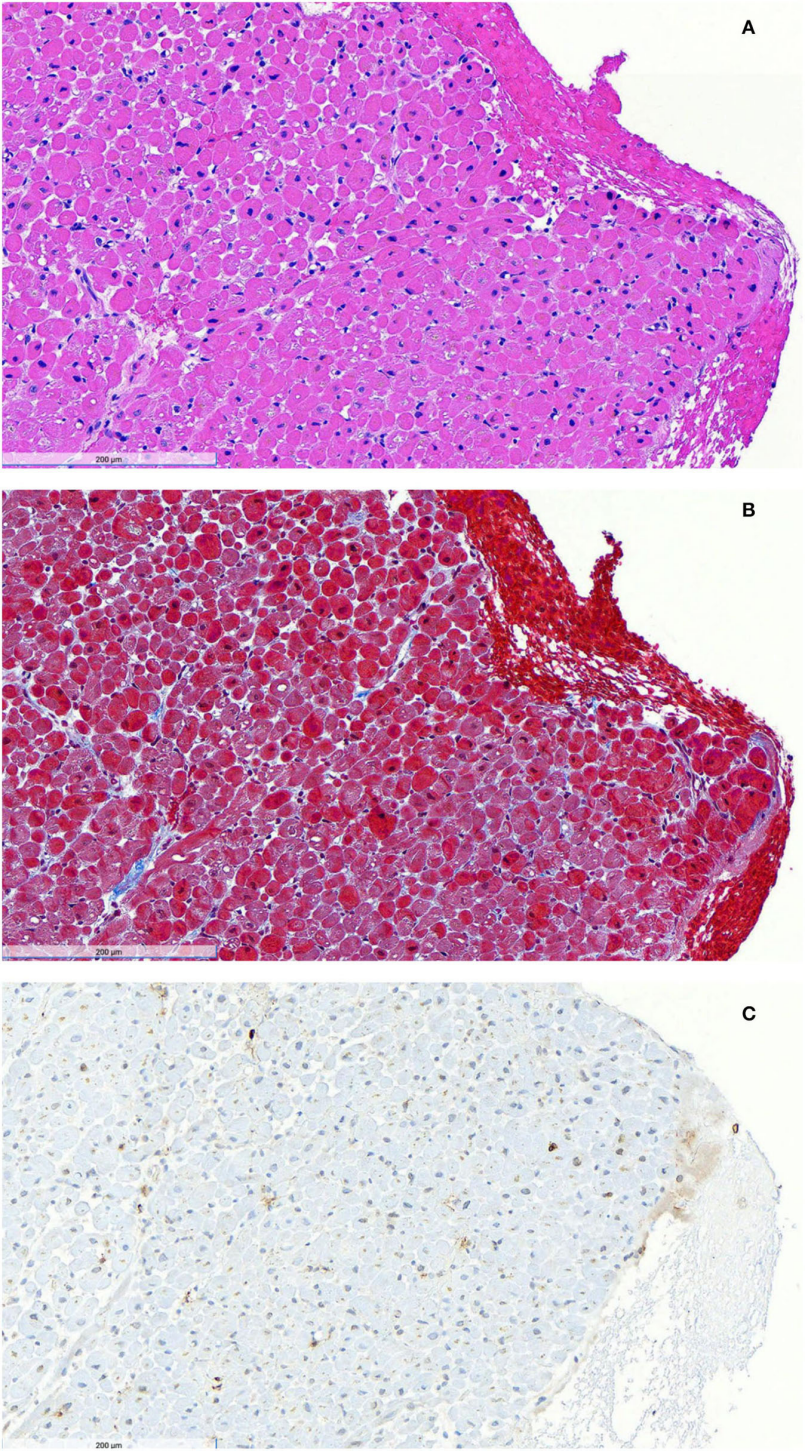
Endomyocardial biopsy after initiation of steroid therapy. Hematoxylin and eosin staining **(A)** shows no acute myocyte necrosis and no significant interstitial expansion. No granulomas or giant cells were observed. Masson trichrome staining **(B)** shows focal mild blue-stained fibrosis. Immunoperoxidase staining for CD3 **(C)** reveals a slight infiltrate of brown CD3-positive T lymphocytes. Immunoperoxidase staining for CD68 demonstrated a diffuse mild infiltrate of CD68-positive macrophages. There was no evidence of amyloid on Congo red stain, no endocardial fibroelastosis on Movat stain, no iron deposition on Fe stain, and no excessive glycogen deposition on PAS stain. Toluidine blue-stained EM thick sections showed mild endocardial fibrosis and some endocardial lipid droplets.

On Day 7 of presentation, the patient was clinically deteriorating rapidly. Interventional cardiology and cardiothoracic surgery were consulted to plan for possible impella or ECMO placement. Her blood vessels were deemed too small for impella or peripheral ECMO. Given the absence of evidence of infection and the lack of improvement with broad-spectrum antibiotics, a diagnosis of cytokine storm syndrome (14) was made, and she was given 1 gram of methylprednisolone daily. Over the ensuing 48 h, her fever resolved, the heart rate decreased from 130 to 90 bpm, CO improved to 4.7 L/min, CI improved to 2.7 L/min/m^2^, SVRI improved to 1,528 dynes-s/cm^5^/m^2^, mixed venous oxygen saturation improved to 57%, and lactic acidosis resolved. ESR decreased to 30 mm/h, CRP decreased to 3.7 mg/dL, IL-6 decreased to 7.9 pg/ml, and IL-2 receptor decreased to 2,217 pg/ml. Her LVOT obstruction and phenylephrine requirement were resolved. Forty-eight hours after initiation of steroids, she underwent an endomyocardial biopsy that showed mild focal fibrosis, a diffuse mild infiltrate of macrophages, and a slight infiltrate of T lymphocytes, without necrosis (Figure 1). Cardiac MRI performed on Hospital Day 12, five days after steroids were initiated, and after LVOT obstruction resolved, showed circumferential hypokinesis of the distal mid-cavity to the apex of the left ventricle with hyperdynamic base maintaining normal function, without fibrosis or edema (Supplementary Video 1). After 3 days of high-dose steroids, she has transitioned to prednisone 40 mg daily, a beta-blocker, and was discharged with a 2-week steroid taper to null.

The patient represented one day after completing the taper with acute onset of pleuritic, left-sided chest and shoulder pain. She was hypotensive and febrile. CRP and soluble IL-2 receptor were again elevated (13.2 from 3.7 mg/dL at discharge and 2,643 from 2,217 pg/ml at discharge, respectively). An echocardiogram showed recrudescence of mild apical hypokinesis. She was treated with high-dose non-steroidal anti-inflammatory drugs and colchicine, with a resolution of her chest pain and improvement of LVEF to 60%.

Four months after her index hospitalization, she was tapered off NSAIDs and remained on colchicine. She was free of further hospitalization, without recurrence of symptoms, and her cardiac function and CRP had completely normalized. Ten months after her index hospitalization, she was taken off colchicine and remained stable, with normal cardiac function and serum CRP.

## Discussion

The case here presented is unique because it highlights the potential role of inflammation as a trigger for a biphasic presentation of Takotsubo cardiomyopathy, it highlights the potential role of pulse dose steroids for the treatment of cytokine storm with hemodynamic compromise, and it adds to the growing body of evidence implicating cytokine storm as a potential trigger for Takotsubo cardiomyopathy.

Cytokine storm is a clinical diagnosis with poorly defined parameters (14). However, the presentation of our patient with fevers, elevated ESR, CRP, and IL-6 in the context of rapid development of lactic acidosis and hemodynamic compromise clearly fits within this presentation. The literature reports several cases of Takotsubo presenting in association with a systemic inflammatory response syndrome, (13) and it is well-known that patients with Takotsubo can have recurrences at variable times after the initial presentation (15). However, we are not aware of prior reports of a “back-to-back” recrudescence of Takotsubo associated with a systemic inflammatory response.

After making a diagnosis of cytokine storm, we decided to treat with pulse dose steroids. Treatment of cytokine storm has not been codified. Current trends point to the use of biologics targeting single cytokines (14). However, pulse-dose steroids are a safe, cheap, and accessible tool to rapidly suppress both the adaptive and innate immune response (16). Treatment response in a single patient cannot prove a cause-and-effect relationship between the administration of high-dose steroids and the rapid clinical improvement that we observed. However, our observation suggests that pulse-dose steroids might be a reasonable option for the treatment of cytokine storm-associated hemodynamic compromise. At least two other cases exist in the literature where steroids were used to treat Takotsubo cardiomyopathy, with good outcomes. One patient was treated for Takotsubo associated with acute demyelinating encephalomyelitis, with improvement in LVEF in 1 week (17), and another was treated for Takotsubo associated with an allergic reaction to dimethyl fumarate (used to treat relapsing-remitting multiple sclerosis), with improvement in LVEF by 3 months (18). While neither of these cases involved treatment of cytokine storm or associated obstructive shock as in our patient, they further support that steroid therapy might be a safe and effective means of treating inflammation-associated Takotsubo.

In our patient, we did not observe myocardial edema on cardiac MRI. This was somewhat unexpected because myocardial edema has been previously reported in Takotsubo (6). However, since our patient completed a cardiac MRI after the completion of 5 days of treatment with high-dose steroids, we speculate that myocardial edema might have subsided in response to treatment.

An additional interesting aspect of the presented case is that our patient developed recurrence of symptoms and pericarditis chest pain after completion of a slow steroid taper. Scally et al. have described systemic inflammation up to 5 months after initial presentation, with both symptoms and myocardial dysfunction up to 20 months after diagnosis (3, 5). Our patient was able to discontinue anti-inflammatory medications after a second slow taper of steroids associated with a prolonged treatment course with colchicine. This suggests that her initial presentation was associated with a degree of chronic inflammation that resolved over several months.

Interestingly, we were unable to find a definitive emotional stressor as the cause of this patient's Takotsubo syndrome. She denied any emotional disturbance during the grandchild's baseball game. However, she did have a preceding migraine, which has been reported as the trigger for the acute onset of Takotsubo stress cardiomyopathy in other patients (19).

In summary, the case here presented is unique insofar as it contributes to the growing appreciation for the role of systemic inflammation in the pathogenesis and evolution of Takotsubo stress cardiomyopathy, and it highlights the potential role of steroids in the treatment of life-threatening hemodynamic compromise secondary to Takotsubo, as well as the potential value of anti-inflammatory therapy to prevent relapse. Dedicated studies will be needed to understand whether the experience from this case can be generalized to a wider patient population.

## Data Availability Statement

The original contributions presented in the study are included in the article/Supplementary Material, further inquiries can be directed to the corresponding author/s.

## Ethics Statement

Ethical review and approval was not required for the study on human participants in accordance with the local legislation and institutional requirements. The patients/participants provided their written informed consent to participate in this study. Written informed consent was obtained from the individual(s) for the publication of any potentially identifiable images or data included in this article.

## Author Contributions

BG, MG, RV, OC, and LA contributed equally and substantially in the conception of the work, in the acquisition, analysis and interpretation of data, in drafting and revision, provided approval for publication of the content, and agree to be accountable for all aspects of the work in ensuring that questions related to the accuracy or integrity of any part of the work are appropriately investigated and resolved. All authors contributed to the article and approved the submitted version.

## Funding

LA is the recipient of NHLBI grants 5K08HLO145108-03 and 1R01HL160716-01.

## Conflict of Interest

LA is a co-founder of i-Cordis, LLC, a company focused on the development of immunomodulatory small molecules for the treatment of heart failure. The remaining authors declare that the research was conducted in the absence of any commercial or financial relationships that could be construed as a potential conflict of interest.

## Publisher's Note

All claims expressed in this article are solely those of the authors and do not necessarily represent those of their affiliated organizations, or those of the publisher, the editors and the reviewers. Any product that may be evaluated in this article, or claim that may be made by its manufacturer, is not guaranteed or endorsed by the publisher.

## Supplementary Material

Supplementary Video 1 Apical 4-chamber cine cardiac magnetic resonance imaging after initiation of steroid therapy. Circumferential hypokinesis of the distal mid-cavity to apex of the left ventricle is observed with the hyperdynamic base maintaining normal function. LVEF, 64%. No fibrosis or edema was observed. Additionally, small pericardial and bilateral pleural effusions with compressive atelectasis are seen.

## Abbreviations

CI: Cardiac index
CO: Cardiac output
CRP: C-reactive protein
ESR: Erythrocyte sedimentation rate
IL-2: Interleukin-2
IL-6: Interleukin-6
LVEF: Left ventricular ejection fraction
LVOT: Left ventricular outflow tract
RR: Reference range.
